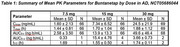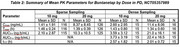# Pharmacokinetic characterization of buntanetap in plasma of patients with early Alzheimer’s and Parkinson’s diseases

**DOI:** 10.1002/alz70861_108862

**Published:** 2025-12-23

**Authors:** Matthew J Peterson, Alexander Morin, Cheng Fang, Maria L. Maccecchini

**Affiliations:** ^1^ Annovis Bio, Malvern, PA USA

## Abstract

**Background:**

Buntanetap (formerly Posiphen) is an orally bioavailable small molecule that improves cognitive function in patients with early Alzheimer’s (AD) and Parkinson’s (PD) diseases. Buntanetap is lipophilic and has high blood‐brain barrier penetrability and brain uptake (10:1 brain:plasma ratio). In SAD and MAD studies, at all doses, buntanetap showed similar profiles, it was absorbed rapidly and cleared from the circulation bi‐phasically, independent of dose. This analysis aims to further evaluate the pharmacokinetics (PK) of buntanetap in AD and PD populations.

**Method:**

The following parameters were measured across two RCTs for buntanetap doses ranging between 7.5mg‐30mg: C_max_, T_max_, AUC_0‐t_, AUC_0‐∞_, t_1/2_. In AD (NCT05686044, Phase II/III, N=351), PK samples were obtained at 0h and then 1h, 2h, and 4h after drug administration (0, 7.5mg, 15 mg, 30mg QD). In PD (NCT05357989, Phase III, N=523), two cohorts of PK samples were obtained: sparse and dense (0, 10 mg, 20mg QD). In the sparse group, PK samples were obtained at 0h and then 1.5h after drug administration. In the dense group, PK samples were obtained at 0h and then 1h, 1.5h, and 2h‐10h (hourly) after drug administration.

**Result:**

Calculated PK parameters for buntanetap in plasma for AD (Table 1) and PD patients (Table 2) had many similarities. C_max_ and AUC_0‐t_ for AD and PD (dense) increased at the same rate in a dose‐proportional manner; PD (sparse) increased with dose as well, but its response was more muted overall. Mean T_max_ ranged from 1.34‐1.88h across all doses in both AD and PD patients. Mean t_1/2_ was comparable and ranged from 1.55‐1.74h in AD and 2.01‐2.57h in PD. The 30mg dose in AD did not follow the increasing trend for AUC_0‐∞_ as in the lower doses for AD and PD (dense), which was likely underpowered by the sampling of this measure (*N* =2).

**Conclusion:**

Analyses from two RCTs confirmed similar mean C_max_, T_max_, and AUC_0‐t_ across doses of 7.5mg‐30mg buntanetap in both patients with early AD and PD. These data confirm fast plasma peak, as seen in all of our previous studies, and dose‐dependent exposure to drug, supporting buntanetap’s PK uniformity across AD and PD indications.